# The “Phantom Effect” of the Rexinoid LG100754: Structural and Functional Insights

**DOI:** 10.1371/journal.pone.0015119

**Published:** 2010-11-30

**Authors:** Yoshiteru Sato, Nick Ramalanjaona, Tiphaine Huet, Noelle Potier, Judit Osz, Pierre Antony, Carole Peluso-Iltis, Pierre Poussin-Courmontagne, Eric Ennifar, Yves Mély, Annick Dejaegere, Dino Moras, Natacha Rochel

**Affiliations:** 1 Département de Biologie et de Génomique Structurales, Institut de Génétique et de Biologie Moléculaire et Cellulaire (IGBMC), Institut National de Santé et de Recherche Médicale (INSERM) U964/Centre National de Recherche Scientifique (CNRS) UMR 1704/Université de Strasbourg, Illkirch, France; 2 Plate-forme technologique de Biologie et Génomique structurales, Institut de Génétique et de Biologie Moléculaire et Cellulaire (IGBMC), Institut National de Santé et de Recherche Médicale (INSERM) U964/Centre National de Recherche Scientifique (CNRS) UMR 1704/Université de Strasbourg, Illkirch, France; 3 Laboratoire de Biophotonique et Pharmacologie, Faculté de Pharmacie, UMR 7213 du CNRS, Université de Strasbourg, Illkirch, France; 4 Institut de Chimie LC3 - CNRS- UMR 7177, ISIS, Strasbourg, France; 5 Architecture et réactivité de l'ARN, Université de Strasbourg, CNRS, Institut de Biologie Moléculaire et Cellulaire, Strasbourg, France; University of South Florida College of Medicine, United States of America

## Abstract

Retinoic acid receptors (RARs) and Retinoid X nuclear receptors (RXRs) are ligand-dependent transcriptional modulators that execute their biological action through the generation of functional heterodimers. RXR acts as an obligate dimer partner in many signalling pathways, gene regulation by rexinoids depending on the liganded state of the specific heterodimeric partner. To address the question of the effect of rexinoid antagonists on RAR/RXR function, we solved the crystal structure of the heterodimer formed by the ligand binding domain (LBD) of the RARα bound to its natural agonist ligand (*all-trans* retinoic acid, *at*RA) and RXRα bound to a rexinoid antagonist (LG100754). We observed that RARα exhibits the canonical agonist conformation and RXRα an antagonist one with the C-terminal H12 flipping out to the solvent. Examination of the protein-LG100754 interactions reveals that its propoxy group sterically prevents the H12 associating with the LBD, without affecting the dimerization or the active conformation of RAR. Although LG100754 has been reported to act as a ‘phantom ligand’ activating RAR in a cellular context, our structural data and biochemical assays demonstrate that LG100754 mediates its effect as a full RXR antagonist. Finally we show that the ‘phantom ligand effect’ of the LG100754 is due to a direct binding of the ligand to RAR that stabilizes coactivator interactions thus accounting for the observed transcriptional activation of RAR/RXR.

## Introduction

Upon ligation to their cognate receptors, naturally-occurring vitamin A derivates mediate several physiological processes, such as vertebrate morphogenesis, cellular growth, differentiation or survival, as well as pathological conditions e.g premature birth, skin diseases or cancer development (reviewed in [1]). The *at*RA isomer binds exclusively to RARs whereas the 9-*cis* form of RA binds to both RARs and RXRs, (each of which exists as three isoforms α, β and γ) (reviewed in [2]).

The ability of RAR/RXR to modulate the expression of target genes results from a combinatorial, coordinated and sequentially orchestrated exchange between nuclear hormone receptors (NHRs) and their coregulators. A general model of RAR/RXR-mediated transcription proposes that unliganded RAR/RXR heterodimers are bound to regulatory elements of their target genes and interact with transcriptional repressor complexes such as NCOR/SMRT/SIN3 to recruit histone deacetylases that lead to repression of target gene transcription [3]. Binding of agonist ligand to the nuclear receptor, triggers a conformational change in the ligand binding domain (LBD) with the repositioning of the C-terminal helix H12 creating a binding surface that allow coactivator to bind. Coactivator proteins such as CBP/p300, the p160 family, CARM1 or the Mediator contain one or more consensus LXXLL motifs that form an α-helix that fits into the hydrophobic cleft on the LBD to allow activation of target genes [4]. Antagonist ligands that prevent the C-terminal helix H12 from adopting its active conformation facilitate the interactions with corepressors.

A significant feature of RXR is its ability to act on its own or in concert with other signalling pathways to induce cell differentiation or apoptosis, as exemplified in immature human promyelocytic NB4 cells [5]. RXR ligands (rexinoids)-mediated biological outcomes depend on the nature and the liganded state of the heterodimeric partner. As such, RAR/RXR is a non-permissive heterodimer in which RAR agonists can autonomously activate transcription while full responses to rexinoids occur only in the presence of RAR agonist ligands [6]–[7]. In addition, RXR ligands are able to bind to the heterodimer even in the absence of RAR ligand [8]–[9]. In sharp contrast, the permissive heterodimers exemplified by PPAR/RXR or Nurr1/RXR are activated by RXRs ligands *per se* [10], [11].

**Figure 1:**
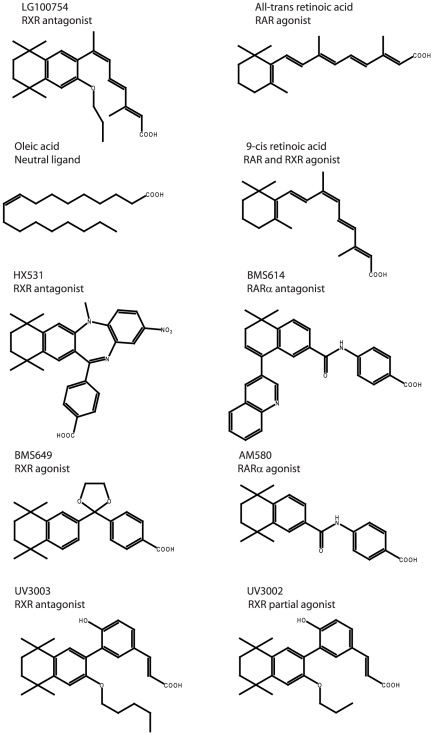
Chemical structures of the RAR and RXR ligands used in this study.

For RAR/RXR heterodimers, the crystal structures of the fully agonist and fully antagonist conformations have been reported [12]–[13]. In the later one, the antagonist BMS614 bound to RARα prevents the positioning of the active conformation of H12 that occludes the coactivator binding site. We now report the crystal structure of the heterodimer formed by the ligand binding domains of the human RARα bound to an agonist (*all-trans* retinoic acid, *at*RA) and the mouse RXRα bound to a rexinoid antagonist, LG100754 (Figure 1) that has been shown to be an antagonist for RXR homodimer and a selective heterodimer antagonist [14]. Although LG100754 activates the PPARα/RXR heterodimer [15], it does not activate TR/RXR, VDR/RXR or LXR/RXR [14]. For RAR/RXR, it has been shown that the binding of LG100754 leads to transactivation mediated by RAR [16]. This effect has been termed the “phantom effect” and the proposed explanation is a conformational change in RAR induced by the binding of LG100754 to RXR promotes SRC-1 coactivator recruitment and transactivation activation via RAR [14]. However a gene expression activation study has shown that LG100754 acts as an antagonist also for the RAR/RXR heterodimer [17]. LG100754 was also shown to be unable to release the corepressor SMRT from RAR *in vitro* [14], but to stimulate release of corepressors in an *in vivo* study [16]. This discrepancy in the ability of LG100754 to promote the dissociation of SMRT corepressor bound to RAR arises from a difference in the sensitivity of the two assays that was closer to physiological conditions in the *in vivo* study [16].

This new structure explains the RXR full antagonism activity of LG100754. The comparison with the previous RAR/RXR structures together with biochemical assays of the SRC-1 coactivator peptide recruitment to the heterodimer provides insight into the molecular mechanism of LG100754 action and explains its phantom effect.

## Results and Discussion

### Overall structure of the agonist/antagonist RARα/RXRα LBD heterodimer

**Figure 2:**
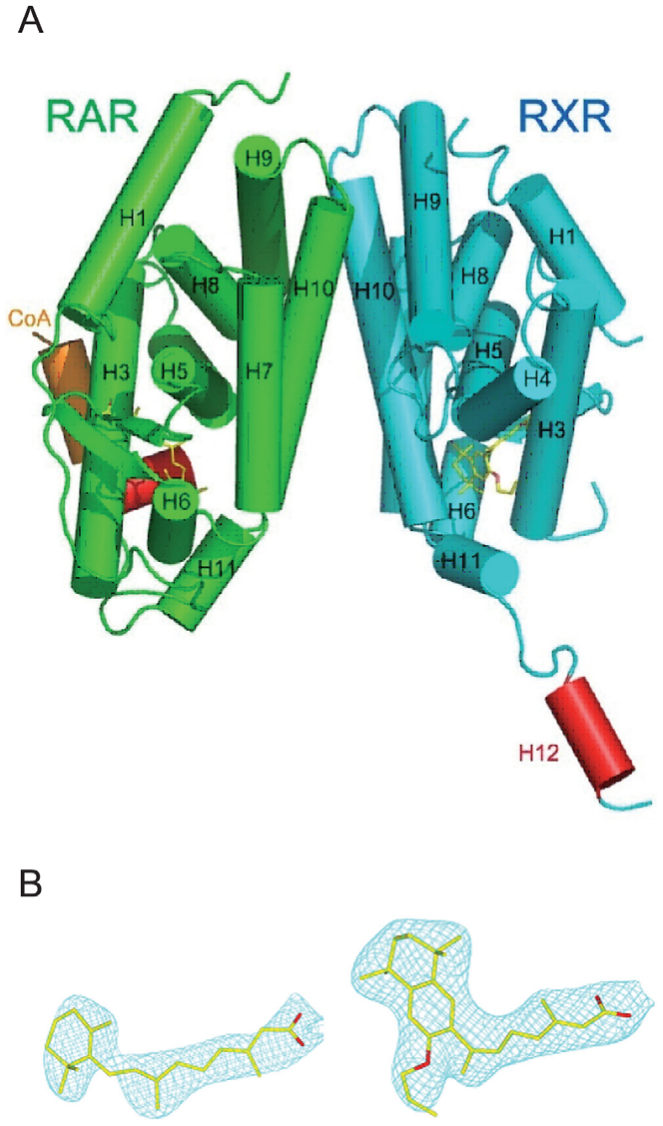
Overall structures of the RARα-*at*RA/RXRα-LG100754 LBD heterodimer. (**A**)The RARα (in green)/RXRα (in cyan) heterodimer is shown by the cylindrical helices representation. Helices are numbered from N- to C-terminus with the activation helices H12 in red. The TIF-2 coactivator peptide bound to RARα through a surface formed by H3, H4 and H12 is shown in orange. The two ligands are shown by stick representation with carbon and oxygen atoms colored in yellow and red, respectively. (**B**) **Conformations of the bound ligands.** *at*RA (left) and LG100754 (right) are shown in their 2*Fo* – *Fc* electron density map contoured at 1.0 σ.

**Figure 3:**
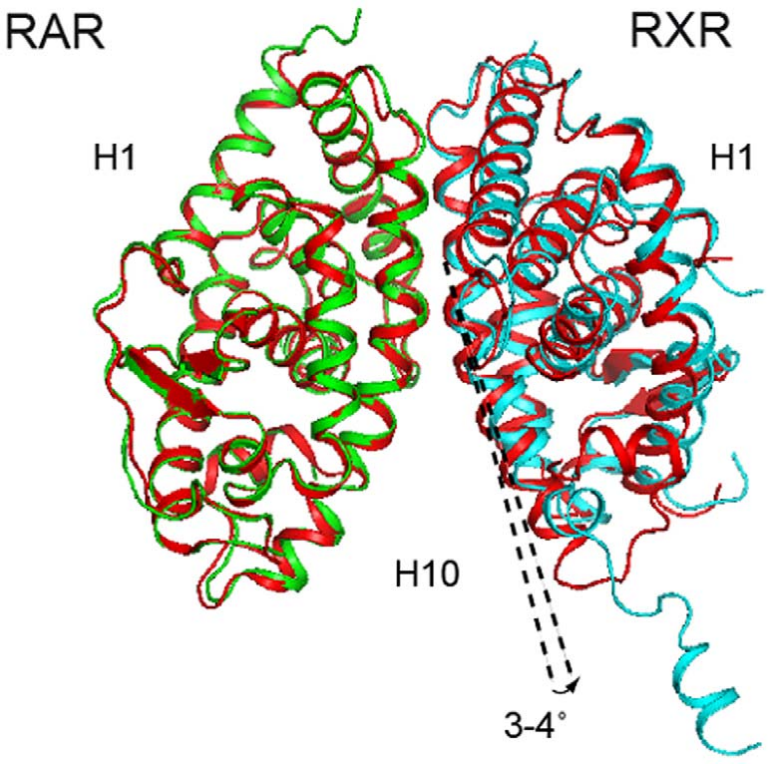
Structural comparison of the RARα-atRA/RXRα-LG100754 LBD heterodimer with the fully agonists RARβ-*9-cis*RA/RXRα-*9-cis*RA heterodimer (PDB ID: 1XDK) showing a rigid body movement of RXR. The RARs are superimposed. The same color code as previously is used while the fully agonists heterodimer is shown in red.

The crystal structure of the ternary molecular complex of RARα-*at*RA/RXRα-LG100754 LBDs and TIF-2 coactivator peptide was solved at 2.75 Å resolution and contains the two receptor LBDs bound to their respective ligands. One TIF-2 coactivator peptide is bound to RARα (Figure 2A). Both LBDs adopt the canonical fold of NR LBDs and are bound to their ligands as shown by the experimental 2Fo-Fc density map (Figure 2B). The present structure adopts an asymmetric agonist/antagonist conformation. Indeed, RARα bound to *at*RA presents the active agonist conformation with the C-terminal helix H12 sealing the ligand binding cavity and one coactivator peptide bound through its LXXLL motif to the coactivator cleft generated by H3, H4 and H12. On the other side, the RXRα LBD bound to LG100754 adopts an antagonistic conformation with H12 pointing to the solvent and preventing the coactivator peptide from binding to RXR. This antagonistic conformation of H12 is different from that observed in the structure of the RXRα-oleic acid in the fully antagonist RARα/RXRα heterodimer, where H12 of RXRα in complex with oleic acid binds to its own cofactor binding site [12]. In addition, we previously reported the crystal structure of an asymmetric heterodimer of the complex of ecdysone receptor (EcR) and ultraspiracle (USP), with EcR in an active agonist conformation and USP in an antagonist one [18]. While in this latter structure, USP forms constitutively an antagonistic conformation, with an inactive locked H12, in the present one, the antagonist conformation is induced by the ligand. Compared with the previous two RAR/RXR structures (PDB IDs are 1DKF and 1XDK), the overall structures are similar to each other except for H11 and H12. The root mean square deviation (RMSD) between RARα-*at*RA/RXRα-LG100754 and the fully agonists RARβ/RXRα is 0.85 Å over the Cα atoms (residues 185–415 for RARα, 178-408 for RARβ and 231–248, 268–440 for RXRα). However, the present heterodimer arrangement shows rigid body movement of RXRα LBD compared with the previous heterodimers with a rotation by 3–4° from the C2 symmetry axis of the dimer, although the overall structures of each RARs and RXRs are similar to each other (Figure 3). The structure superposition of the monomeric RAR and RXR LBDs onto their corresponding heterodimers shows little variations between monomeric and heterodimeric receptors with RMSDs of 0.55 Å between the RARα-*at*RA and the RARβ-9-*cis*RA, and 0.54 Å between the RXRα-LG100754 and the RXRα-9-*cis*RA.

### Solution structure determination of RARα-atRA/RXRα-LG100754

**Figure 4:**
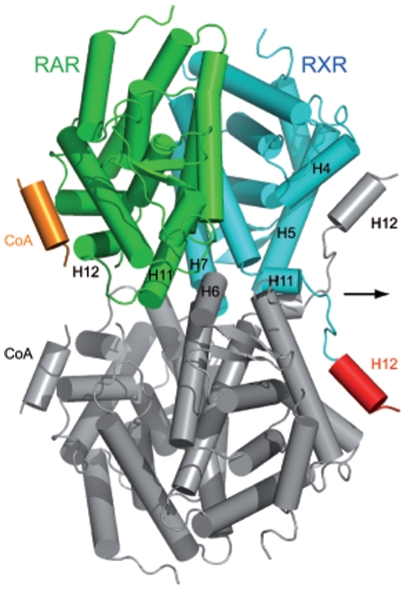
Crystal packing of the agonist/antagonist RAR/RXR LBD heterodimer according to the crystallographic 2-fold symmetry. The same color code as previously was used. The crystallographic 2-fold symmetry related heterodimer is shown in gray. Helices which are involved in the packing are labelled.

The crystal packing induces an intermolecular interaction between the flipped H12 of the RXRα-LG100754 and the coactivator binding surface of a symmetry related RXR molecule (Figure 4). This packing interface is made not only by the cofactor binding site but also H11 of RAR, H6, LoopH6-H7, H7 and H11 of RXR (Figure 4). Total surface area buried between the packing interface is 2000 Å^2^. Such tetrameric assembly induced by the flipped H12 is also observed in the other crystal structures of RXR [PDB IDs: 1LBD, 1G5Y, 1H9U, 2Q60, 2GL8] but the orientation of the present tetramer is different from any other. The oligomeric state of this complex in solution was determined by Small Angle X-ray Scattering (SAXS) the values of the radius of gyration R_g_ and of the maximal dimension D_max_ as structural parameters (Table 1 and Figure S1). We further compared them to those of other RAR/RXR heterodimer or RXR homodimer or tetramer [19] and to theoretical values calculated from crystallographic structures. The values of R_g_ and of D_max_ parameters measured for the RARα-*at*RA/RXRα-LG100754 complex clearly indicate that the complex is dimeric in solution. The R_g_ is 5 Å smaller than that calculated from the tetrameric crystal structure (Figure S1). Furthermore, the best fit of the experimental data is obtained unambiguously with the dimeric model in which one monomer is in a closed conformation and the other one in an open conformation with helix H12 pointing to the solvent (Figure S1). The experimental R_g_ is 1 Å larger than that of the heterodimer fully bound to agonists indicating a less compact conformation and 1 Å smaller than the relaxed apo-form of RXR dimer (Table 1). The tetramer generated by the crystallographic symmetry is induced by the highly concentrated conditions during the crystallization process.

**Table 1:** Small angle X-ray parameters.

Complexes	R_g_ (Å)	D_max_ (Å)	R_g_theo(Å)	χ
RAR-9-*cisRA*/RXR-*9-cis*RA	26.0±0.2	87±5		
RAR-*at*RA/RXR-LG100754	27.2±0.2	90±5		
RXR/RXR-*9-cis*RA [19]	26.7±0.3	80±5		
apo RXR/RXR [19]	28.0±0.3	92±5		
apoRXR/RXR tetramer [19]	32.8±0.3	95±5		
RAR-*at*RA/RXR-LG100754 dimer			26.4	1.8
RAR-*at*RA/RXR-LG100754 tetramer			32.1	8.9
RXR/RAR-*9-cis*RA (1XDK)			26.0	2.6
RAR-BMS614/RXR-oleic acid (1DKF)			26.1	2.5

R_g_ and D_max_ are the radius of gyration and maximum size, respectively computed from the entire scattering pattern using the indirect transform package GNOM [56]. R_g_theo is the radius of gyration calculated from the crystal structure using CRYSOL [57]. Discrepancy between the experimental data and the scattering curves calculated from the crystal structures is denoted as χ.

### Dimer interface analysis of the RARα-atRA/RXRα-LG100754

The heterodimer interface is made by H7 (353–361), H9 (395–410) and H10-H11 (417–435) of RXR, and LoopH8-H9 (336–340), H9 (349–364), H10 (371–380) of RAR and is similar to those of the previously solved RAR/RXR heterodimers. The comparison of the heterodimer interface (Figure 3) between the present asymmetric RARα/RXRα heterodimer, with the fully agonist RARα/RXRα and the fully antagonist RARα/RXRα heterodimers, using free energy decomposition analysis [20] showed that the important interactions stabilizing the dimer are conserved. The rigid body movement of RXRα LBD in the present RARα/RXRα heterodimer caused a small reduction in the interface area, but the salt bridges remained conserved (shown in Figure S2). The intramolecular salt bridges which are specific to class I NHR LBDs namely Glu244-Arg376 and Glu371-Arg419 for RXRα, and to class II NHR LBDs, namely Asp267-Arg339 and Glu325-Arg367 for RAR are maintained [21]. The conformation and surrounding network of Tyr402 of RXR which plays an important role for dimerization [22] are also conserved.

### Ligand binding mode in RARα-atRA/RXRα-LG100754

The binding mode of *at*RA (Figure 2B) to RARα in the present heterodimer is identical to that observed previously for RARα LBD [23]. The size of the *at*RA is 278 Å^3^. The comparison of the volume of the ligand binding cavity is 418 and 503 Å^3^ and the retinoic acid occupies 66.5% and 55.3% of the pockets for RARα and RARα, respectively. The difference of the cavity size around 100 Å^3^ is due to the different residues of the two isotypes forming the ligand binding pocket (LBP).

**Figure 5:**
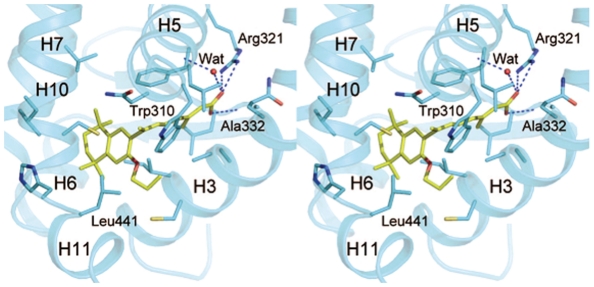
A stereoview of the interactions of LG100754 with the ligand binding pocket of RXRα. Only residues closer than 4.2 Å to the ligand are shown. Hydrogen bonds are shown as dotted lines. The secondary structure of the hRXRα-LBD and specific residues are labelled.

**Figure 6:**
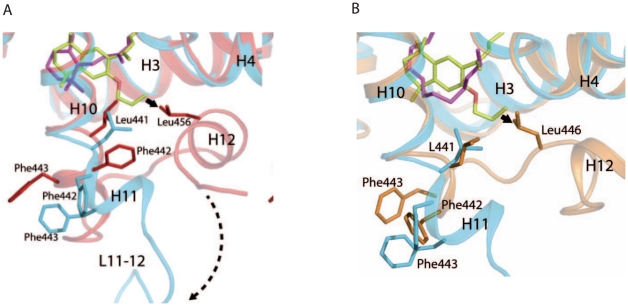
Structural basis of RXR antagonism induced by LG100754. (**A**) Close-up view showing the superposition of RXRα LBP bound to LG100754 (in cyan) and to *9-cis*RA (in red). LG100754 and *9-cis*RA are shown by stick representation in yellow and magenta, respectively, with oxygen atoms in red. The propoxy group of LG100754 induces a steric hindrance (solid arrow) with Leu456 (H12). Residues involved in the transition agonist to antagonist transition (dotted arrow) conformation are labelled. (**B**) Close up view of the superposition of RXRα LBP bound to LG100754 (in cyan) and to oleic acid in antagonist conformation (in orange). Oleic acid is show by stick representation in magenta. The propoxy group of LG100754 induces a steric hindrance (solid arrow) with Leu446 as shown by an arrow.

The rexinoid antagonist LG100754 is buried in the LBP of RXRα formed by residues located on helices 3, 5, 7, 11 and the β-turn (Figure 5). The interactions are mainly hydrophobic with 80 Van Der Waals (VDW) contacts with the LBP at 4.2 Å cutoff. The carboxylate group makes an anchoring salt bridge with Arg321 [hArg316] (H5) and hydrogen bond with amino group of Ala332 [hAla327] (LoopH5-H6) in the hydrophobic pocket, similarly as observed with the carboxylate of 9-*cis*RA in the RXRα complex [24]. One water molecule makes a hydrogen bond network between the carboxyl group of LG100754 and the amino group of Leu314 [hLeu309]. The tetrahydronaphatalene moiety of LG100754 interacts with residues of H3, H5, H7 and H11 through VDW contacts and notably with Trp310 [hTrp305] (H5) (Figure 5). Compared to the 9-*cis*RA-bound RXR, the carboxylate and tetrahydronaphatalene group of LG100754 are located at the places which correspond to that of the carboxylate and β-ionone group of 9-*cis*RA. The propoxy group is pointing towards H11 and interacts with this helix through VDW contacts notably with Leu441 [hLeu436] which is repositioned (Figure 6A). The electron density map of the end of the propoxy group is poor because of its flexibility (see Figure 2B). A remarkable feature is the solvent accessibility of this LBP because of the flip of H12 to the solvent. According to crystallographic symmetry, this accessible region of the LBP is covered by LoopH11-H12 (mainly Phe443 [hPhe338] and Asp449 [hAsp444]) of another RXRα symmetry related molecule (Figure 4). The active agonistic conformation of H12 of RXRα is prevented by the long-tailed propoxy group of LG100754 which induces a steric hindrance with Leu456 [hLeu451], and consequently the coactivator peptide binding as shown for the superimposition of RXR-LG100754 and RXR-9*cis* RA (Figure 6A). Oleic acid, a neutral RXR ligand, has been crystallized in an RXR agonist conformation in RXR homodimer [25] and in an RXR antagonist conformation in RAR/RXR heterodimer [12]. Superposition of RXRα bound to LG100754 and to oleic acid in RXR antagonist conformation shows two different antagonist conformations. Indeed, the propoxy group of LG100754 induces a steric hindrance with Leu446 [hLeu441] in the LoopH11-H12 as observed in the RXR-oleic acid antagonist conformation, precluding H12 binding to the coactivator cleft (Figure 6B). This new structural information is in agreement with the inability of RXRα-LG100754 homodimer to bind to any coactivator or corepressor [26]. Phe442 [hPhe437] and Phe443 [hPhe438] in H11 of RXRα which are known to play important roles in the transition of the apo to agonist conformation [13], flip out to the solvent region in the present antagonistic structure (Figure 6).

### Structural comparison of LG100754 with other RXR antagonists

Among the few reported RXR antagonists [27]–[28], two other types have been described, namely the dibenzodiazepine derivative HX531 [29] and UVI3003 [30] (Figure 1). In the first case, a docking model proposed [24] that the additional bulky NO_2_ group of HX531 causes a steric hindrance with Gln311 [hGln306] (H5), Trp310 [hTrp305] (H5) and Leu438 [hLeu433] (H10). Indeed, a different antagonistic structure should result in different action on coregulator interaction and function of RXR. Since Leu438 [hLeu433] is part of the dimerization interface, the steric hindrance with Leu438 [hLeu433] is likely to affect the dimerization.

In contrast, the structural basis of the antagonism of UVI3003 should be similar to that of LG100754. The crystal structure of the complex of RXR and the partial agonist UVI3002 [30] (Figure 1) reveals that the alkyl ether group of UVI3002 is located at the same position as the propoxy group of LG100754 but its length do not prevent the agonist conformation. Therefore, UVI3003 which has a longer alkyl group than UVI3002 should similarly prevent H12 associating to the LBD and the RXR complex should adopt an antagonistic conformation as in RXR-LG100754. In agreement with this molecular mechanism of antagonism, analogues of LG100754 with shorter groups such as ethyl or methyl groups instead of the propoxy group act either as partial agonist or full agonist for RXR, respectively [31].

A recent NMR study on the effect of RXR antagonists on the conformation of H12 in the RXR homodimer and in the permissive PPAR/RXR with PPAR bound to an agonist ligand reveals similar features, namely the rexinoid antagonist is unable to stabilize a compact state and therefore prevents the coactivator from binding to RXR [32].

### LG100754 has no effect on the cross-talk between LBD partners of RAR/RXR

**Figure 7:**
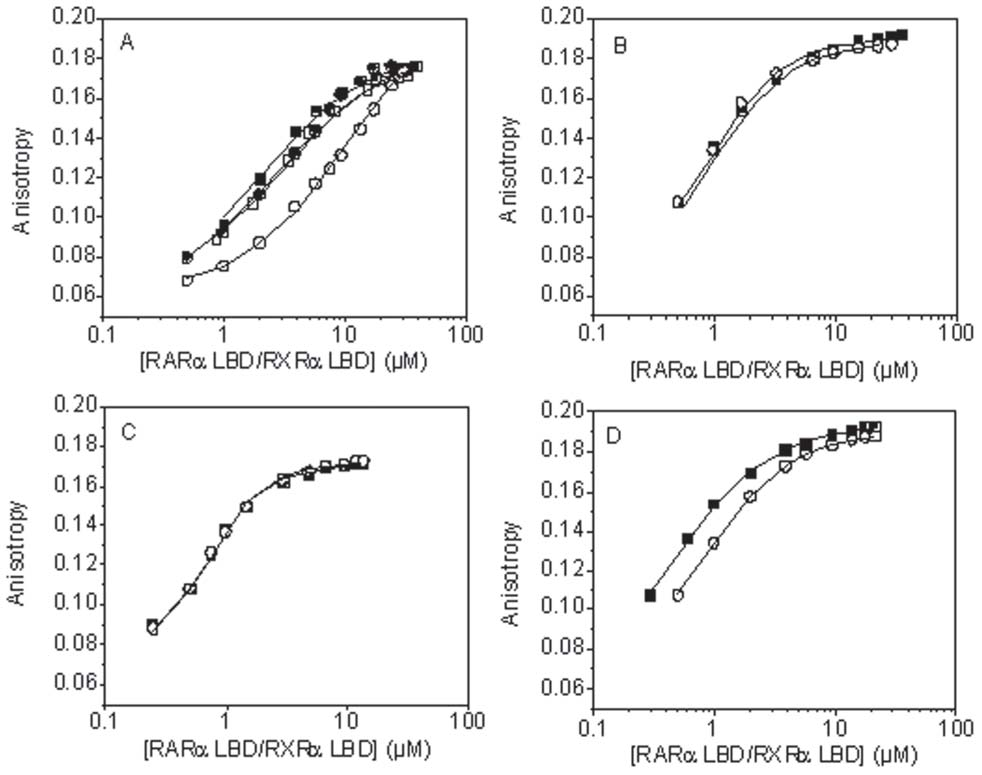
Titration of the TAMRA-labeled SRC-1 NR2 peptide with RARα/RXRα LBD bound with retinoid agonists or antagonists, as monitored by fluorescence anisotropy. (**A**) Titration with the apo-heterodimer (▪) or with the heterodimer bound to LG100754 (□), HX531 (•) or a BMS614/LG100754 combination (○). (**B**) Titration with the heterodimer bound to BMS649 (▪) or a BMS614/BMS649 combination (○). (**C**) Titration with the heterodimer bound to AM580 (○) or AM580/LG100754 combination (▪). (**D**) Titration with the heterodimer bound to *9-cis*RA (○) or a AM580/BMS649 combination (▪). The concentration of TAMRA-labeled SRC-1 NR2 peptide is 1 µM for experiments reported in (B–D).

As LG100754 triggers no effect on RAR structure within the heterodimer, we decided to characterize the ligand effect on the cross-talk between the LBD partners by monitoring the binding of fluorescently labelled SRC-1 NR2 peptide to the heterodimer using fluorescence anisotropy (Figure 7 and Table 2). In its apo-form, the heterodimer was found to bind the SRC-1 NR2 peptide with a 1∶1 stoichiometry and a k_d_ value in agreement with the literature [13]. The binding of LG100754 in a concentration sufficient to saturate only one site and in absence of RAR ligand modifies neither the binding stoichiometry nor the heterodimer affinity for the SRC-1 NR2 peptide (Table 2), in variance with the “phantom ligand effect” hypothesis, where the binding of LG100754 to RXR was thought to affect the cofactor recruitment by its apo-partner [16]. Finally, we repeated the titration in the presence of both LG100754 and BMS614, a specific RARα antagonist [9]. In this condition, the heterodimer binds to the SRC-1 NR2 peptide with a strongly reduced affinity (k_d_>10 µM), confirming that RARα LBD is the main binding site for the SRC-1 NR2 peptide [13]. Taken together, these data show that antagonists bound to RXRα do not affect the binding of the SRC-1 NR2 peptide to the RARα apo-form.

**Table 2:** Binding constants of the SRC-1 NR2 peptide to the RARα/RXRα LBD heterodimer.

RAR ligands	RXR ligands	n	kd (µM)
apo	apo	1	2±0.1; 3±0.6^c^
apo	LG100754	1	2.5±0.1
apo	BMS649	1	0.42±0.03
AM580	apo	1	0.19±0.02
AM580	LG100754	1	0.17±0.01
BMS614	BMS649	1	0.45±0.04
BMS614	LG100754	1^a^	>10^a^
9-*cis*RA	9-*cis*RA	2	(0.61±0.02)^b^
AM580	BMS649	2	(0.29±0.02)^b^

Experiments were carried out by fluorescence anisotropy using the TAMRA-labeled SRC-1 NR2 peptide in a 10 mM TRIS-HCl (pH 7.5), 150 mM NaCl, 10 mM DTT.

a The binding stoichiometry could not be determined in this case, due to the too low affinity.

b Fitting the experimental points systematically provided a k2 value identical to that of k1, indicating that the two dissociation constants are very close to each other.

c from reference [13].

In contrast, a significant increase in the binding constant (k_d_ = 0.42±0.03 µM) of the SRC-1 NR2 peptide for the heterodimer was observed in the presence of the rexinoid agonist BMS649 [33] (Figure 1). Moreover, an identical binding curve (k_d_ = 0.45±0.04 µM) was observed when the titration was performed in the presence of both BMS614 and BMS649 (Figure 7). To check whether a ligand-induced structural change on RARα affects the RXR ability to bind the coactivator peptide, a titration of the labelled SRC-1NR2 peptide by the heterodimer was performed in the presence of the RARα agonist AM580 [34] (Figure 1), with or without LG100754 (Table 2). As expected, AM580 alone enhances the heterodimer affinity for the SRC-1 NR2 peptide (k_d_ = 0.19±0.02 µM), while in conjunction with LG100754 similar results in terms of stoichiometry and binding affinity were seen (k_d_ = 0.17±0.01 µM) (Figure 7), confirming that AM580 promotes the binding of the SRC-1 NR2 peptide to the RARα subunit. The results above clearly show that ligand binding to one subunit of the heterodimer does not positively affect the SRC-1 peptide recruitment by the second subunit, when in its apo-form. Moreover the binding of an antagonist ligand to one subunit does not affect the recruitment of the coactivator peptide on the other one. Binding experiment in the presence of the two agonists ligands AM580 and BMS649 or 9-*cis*RA (Figure 7 and Table 2) indicate a stoechiometry of 2 peptides bound to the heterodimer and that both RARα and RXRα were able to bind the SRC-1 NR2 peptide with almost the same affinity.

### Direct binding to RARα can explain the phantom effect of LG100754

**Figure 8:**
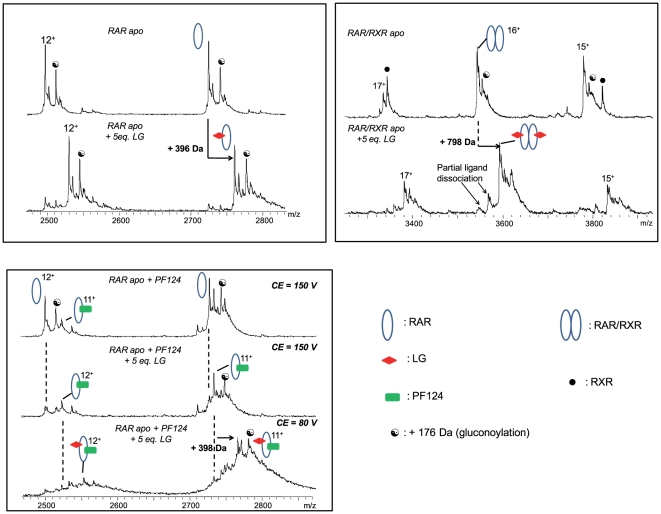
LG100754 binds to both RAR and RXR. (**A**) Enlarged view of the 11+ and 12+ ions of the ESI mass spectrum of RARα in absence (top) and in presence (down) of fivefold molar excess of LG100754 (CE = 80 V). Additional peaks at +60 Da (a) might correspond to acetate adducts. (**B**) ESI mass spectrum of the RARα/RXRα heterodimer in absence (top) and in presence of fivefold molar excess of LG100754 (CE = 80 V) (**C**) Influence of the LG100754 ligand binding on the recruitement of the SRC-1 NR2 peptide by RARα.

To assess whether LG100754 is able to directly bind to RAR, we monitored its interaction with RARα and RARα/RXRα LBD by ESI-MS under non-denaturating conditions. Addition of fivefold molar excess of LG100754 in the RARα LBD resulted in the appearance of a novel series of mass/charge (m/z) ions corresponding to a fully bound RARα-LG100754 complex (Figure 8A). A molecular mass of 30380.4±2.5 Da was obtained which corresponds to the binding of one molecule of LG100754 to the RARα monomer (ΔM = 396 Da). In the RAR/RXR dimer, we mainly observed 2 molecules of ligand bound to the dimer (ΔM = 798 Da) (Figure 8B). In order to detect the protein/ligand complexes, the capillary voltage (CE) which controls the kinetic energy transferred to the ions in the interface region of the mass spectrometer has to be carefully set. Below CE = 80 V, protein/ligand complexes are quantitatively formed; however, the observed peak shapes are broad which probably results from incomplete desolvation. A direct consequence of this is a poor signal/noise ratio and a loss in mass accuracy. Increasing CE to 80 V significantly improves the signal/noise ratio and still allows the detection of the protein/LG100754 complexes as the main component. In contrast to the low binding affinity reported for the binding of LG100754 to RARα [14], we show that this ligand binds to RAR with a significant affinity. The discrepancy can be explained by the low sensitivity of the methods used previously [14]. The use of larger SRC-1 domain as reported [14] instead of short peptide does not explain this discrepancy, as the SRC-1 domain (940-1061 [14]) used didn't contain the nuclear receptor interacting domain (SRC-1 RID 627–786 [35]). We then monitored the effect of the LG100754 on the recruitment of the SRC-1 NR2 coactivator peptide to RARα and RARα/RXRα. In absence of ligand, only a small proportion of RARα is able to bind the SRC-1 NR2 peptide (Figure 8C top). The addition of LG100754 strongly stabilizes the interactions between the RARα monomer and the SRC-1 NR2 peptide since the RARα-LG100754/SRC-1 NR2 complex is now the main species (100%). As described previously for Figures 8A and 8B, decreasing the CE voltage from 150 V to 80 V allows the detection of the intact ternary complex. Interactions involving the ligand are thus less stable than those involving the coactivator peptide in the gas phase. Increasing the CE voltage to 200 V (data not shown) does not lead to any major change in the dissociation pattern regarding the complexes which suggest that the RARα/SRC-1 NR2 complex is quite stable in the gas phase. Note that we have not observed SRC-1 coactivator peptide binding to RXRα-LG100754 (data not shown).

**Figure 9:**
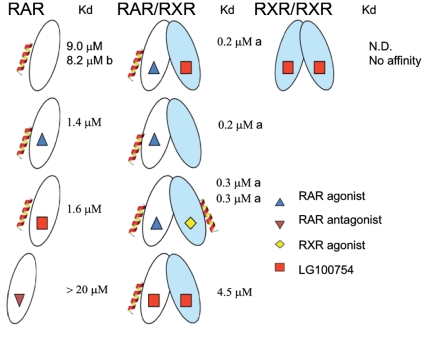
Summary of the effects of ligands on the affinity and stoechiometry of SRC-1 NR2 peptide measured by ITC. ^a^ dissociation constants measured by fluorescence anisotropy. ^b^ from reference [13]. N.D. not determined.

To quantify the recruitment of the SRC-1 NR2 peptide to RARα, RXRα and RARα/RXRα, Isothermal Titration Calorimetry (ITC) was used, thus providing the full thermodynamic profile of SRC-1 binding. The similar SRC-1 NR2 peptide (25 residues) to the one used in ESI-MS was used in the ITC experiments. Representative titrations for SRC-1 NR2 binding are shown in Figure S3. In the presence of LG100754, the RARα monomer binds the SRC-1 NR2 peptide with an affinity similar to that measured in presence of an RAR agonist ligand (Figure 9 and Table S2). Therefore, the LG100754 ligand stabilizes the agonist conformation of RAR that renders accessible the binding surface for coactivator recruitment. Docking of L100754 in the ligand binding pocket of hRARα reveals that the ligand easily adapts to fit the RARα agonist conformation without significant steric clashes (Figure S4). No significant interaction of the SRC-1 NR2 peptide with RXRα LBD was observed (Figure S3) in agreement with our ESI-MS data and with the literature [26]. In RAR/RXR LBDs bound to LG100754, SRC-1 NR2 binds to the heterodimer with a stoechiometry of one peptide per heterodimer and with an affinity similar to the one for the RARα monomer. Together these data demonstrate (Figure 9) that the LG100754 compound is able to stabilize an agonist conformation when bound to RAR while in RXR it inhibits the interaction with the coactivator SRC-1 peptide. LG100754 has also been shown to be able to dissociate corepressors from RAR [16] and to prevent their binding to RXR [26].

### Conclusions

The crystal structure of the RAR-*at*RA/RXR-LG100754 complex revealed that RAR adopts an agonist conformation and RXR an antagonist one. The RXR antagonist LG100754 conformation affects neither the dimerization of RAR/RXR nor the active conformation of RAR. The orientation of the propoxy group of LG100754 prevents H12 to packing against the LBD and the coactivator peptide binding to RXR. A similar antagonist mechanism has been observed in the crystal structure of the ERα LBD bound to the anti-estrogen ICI164384 [36]. We observe that LG100754 induces a high mobility of H12 and acts as a full RXR antagonist. Fluorescence anisotropy titrations showed that the presence of both agonists in the dimer leads to the highest affinity for the coactivator peptide, the binding of an antagonist to one subunit and of LG100754 to RXR does not affect the recruitment of the coactivator by its partner. Our study gives an explanation to the phantom effect of LG100754 that is due to its direct activation through RAR. Our findings provide new insights into the wide spectrum of molecular interactions in the RAR/RXR dimer and for the design of more specific RXR antagonist drugs that have great promise for the prevention and treatment of cancer and metabolic diseases [37].

## Materials and Methods

### Materials and chemicals

LG100754 was kindly provided by MSD (N.V. Organon). 9-*cis* retinoic acid (9-*cis*RA), *at*RA and AM580, a synthetic RAR pan-agonist, were purchased from Sigma. BMS614 and BMS649 were provided by Bristol-Myers Squibb. Fluorescent NR box2 motif Steroid Receptor Coactivator-1 (686-RHKILHRLLQEGS-698) peptide (SRC-1 NR2) was purchased from Neosystem (Strasbourg, France).

### Expression, Purification and Crystallization

The human RARα LBD (residues 153–421) was cloned as an N-terminal hexahistidine-tagged fusion protein in a pET15b expression vector and the mouse RXRα LBD (residues 228–467) was cloned into a pET3a expression vector. Both were produced in an *E. coli* BL21 (DE3) strain. Cells were grown in LB medium for 3 h at 37°C and subsequently induced for 3 h at 20°C with 1 mM isopropyl-β-D-thiogalactopyranoside. The His-RAR/RXR heterodimer was copurified by nickel affinity chromatography and gel filtration. The final protein buffer was Tris-HCl 20 mM (pH 8.0), NaCl 150 mM, DTT 10 mM, and TCEP 2 mM. The protein was concentrated to 3 mg/ml and incubated with a 1.5-fold excess of *at*RA, LG100754 and the TIF-2 coactivator peptide (686-KHKILHRLLQDSS-698) prior to crystallization assays. Purity and homogeneity were assessed by SDS and Native PAGE as well as denaturating and native electrospray ionization mass spectrometry. Crystals of the ternary complexes of hRARα/mRXRα LBDs and TIF-2 peptide were obtained at 17°C by vapor diffusion in hanging drops by mixing of 0.5 µl of the protein solution and 0.5 µl of reservoir solution which contains 200 mM potassium thiocyanate and 20% PEG3350.

### Data Collection, Structure Determination and Refinement

The crystals were mounted in fiber loops and flash-cooled in liquid nitrogen after cryoprotection with the reservoir solution plus 5% ethylene glycol. Of 100 crystals tested, only one diffracted to 2.75 Å. Data collection from the frozen crystal was performed at 100 K on the beamline ID23-1 at the ESRF (Grenoble, France). The crystal belongs to the tetragonal space group P4_3_2_1_2, with one heterodimer per asymmetric unit. The data were integrated and scaled using HKL2000 [38] (statistics in Table S1). The structure was solved by molecular replacement using the program AMoRe [39]. The structure of the antagonist-bound heterodimer (PDB ID: 1DKF) was used as a starting model. Refinement involved iterative cycles of manual building and refinement calculations. The programs CNS [40], REFMAC [41], phenix.refine [42], O [43] and COOT [44] were used throughout structure determination and refinement. Several terminal residues and 13 residues between H1 and H3 of RXR (251-263) are not modelled as the electron density map was poor in the corresponding regions. The TIF-2 peptide and the ligand molecules were only included at the last stage of the refinement. The omit map from the refined atomic model of the heterodimer was used to fit the peptide and ligands to their electron densities (Figure S5). Anisotropic scaling, a bulk solvent correction and TLS restraints were used for the refinement. Seven TLS groups for each LBD and one group for the peptide were generated by using the program TLSMD [45]. Individual atomic B factors were refined isotropically. Solvent molecules were then placed according to unassigned peaks in the electron density map. In the RARα-*at*RA/RXRα-LG100754 LBDs and TIF-2 peptide complex, refined at 2.75 Å with no σ cutoff, the final model contains 239 residues (177–415) for hRARα LBD, 218 residues (230–250, 265–461) for mRXRα LBD, 11 residues for TIF-2 peptide, 2 ligand molecules, and 115 water molecules. According to PROCHECK [46], 91.5% of the peptide lies in the core regions, 8.2% in the allowed regions and 0.2% in the generous region. The peptide classified in the generous region of the Ramachandran plot is around Asp449 of RXR. Since this peptide is located at the loop between H11 and H12, such unfavourable conformation is adapted due to intermolecular interaction imposed on H12 by crystal packing (described below). Data are summarized in Table S1. The volumes of the ligand-binding pockets and ligands were calculated by using the program VOIDOO [47] and GRASP [48], respectively. Structural figures were generated by using the Pymol program [49] and CCP4MG [50].

### Free energy decomposition

To quantify the electrostatic and van der Waals contributions to dimer association, a free energy decomposition analysis was performed on the present structure, as well as on the fully agonists and fully antagonists conformations [12]–[13]. Starting from the crystal structures hydrogen atoms were added using the HBUILD [51] module of the CHARMM (Chemistry at HARvard Macromolecular Mechanics) program [52]. The structures were energy minimized and subsequently used in a MM/PBSA decomposition protocol. Details of the protocol are described in reference [20]. Docking of the ligand LG100754 in the binding pocket of hRARα LBD in agonist conformation was performed with Autodock 4.0 using standard input parameters.

### SAXS experiments and data processing

The synchrotron radiation X-ray scattering data were collected at the storage ring DORIS III of the Deutsches Elektronen Synchrotron (DESY) [53]. The scattering patterns were recorded in the range of momentum transfer 0.15<s<3.5 nm- (s = 4πsin(θ)/λ where 2θ is the scattering angle and λ = 0.15 nm is the X-ray wavelength). All studied complexes were measured for at least three protein concentrations ranging from 2 to 5 mg/ml. To check for radiation damage, the data were collected in 1-minute frames which were averaged and processed using standard procedures by PRIMUS [54]. The forward scattering I(0) and the radii of gyration R_g_ were evaluated using the Guinier approximation [55] assuming that at very small angles (s<1.3/R_g_) the intensity is represented as I(s) = I(0)exp{-(sR_g_)2/3}. These parameters were also computed from the entire scattering pattern using the indirect transform package GNOM [56], which also provides the maximum dimension of the particle D_max_ and the distance distribution function P(r). Theoretical values from crystal structures were calculated with CRYSOL [57].

### Fluorescence anisotropy measurements

Steady-state fluorescence anisotropy measurements were performed with a T-format SLM 8000 spectrofluorometer, thermostated at 20°C. A home-built device ensured the automatic rotation of the excitation polarizer. Anisotropy titrations were carried out by adding increasing hRARα/mRXRα LBDs concentrations to a fixed concentration of tetramethylrhodamine (TAMRA) – SRC-1 NR2 peptide in 10 mM TRIS-HCl (pH 7.5), 150 mM NaCl, 10 mM DTT buffer. The binding stoichiometry was determined at peptide concentrations between 1 µM and 10 µM, while the binding constants were determined at a peptide concentration of 1 µM. The excitation wavelength was 530 nm and the emitted light was monitored through high-pass filters (550 nm, Kodak). The Scatchard equation was rewritten to fit the anisotropy, r, as follows:
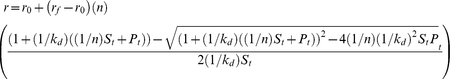
where P_t_ and S_t_, designate the total concentration of the heterodimer and (TAMRA) – SRC-1 peptide, respectively. r_f_ represents the anisotropy at the plateau when all the heterodimer is bound, whereas r_0_ and r correspond to the anisotropy values of (TAMRA) – SRC-1 NR2 in the absence and in the presence of a given concentration of heterodimer, respectively. k_d_ and n correspond to the apparent dissociation constant and the number of binding sites for SRC-1 binding to the heterodimer, respectively. The titration curves were fitted with the Microcal Origin 6.1 software based on the nonlinear, least-squares method and the Levenberg– Marquardt algorithm.

### Electrospray ionization mass spectrometry

Prior to ESI-MS analysis, samples were desalted on Zeba Spin desalting column (Pierce) in 200 mM ammonium acetate (pH 8.0). ESI-MS measurements were performed on an electrospray time-of-flight mass spectrometer (MicrOTOF, Bruker Daltonic, Germany). Purity and homogeneity of the retinoid receptors were verified by mass spectrometry analysis in denaturing conditions: proteins were diluted to 5 pmol/µl in a 1∶1 water-acetonitrile mixture (v/v) acidified with 1% formic acid. Mass spectra were recorded in the positive ion mode after calibration with horse heart myoglobin diluted to 2 pmol/µl in a 1∶1 water-acetonitrile mixture (v/v) acidified with 1% formic acid. The following molecular weights were measured: 29983.6±1.6 Da for RARα and 30159.9±2.2 Da corresponding to an additional covalent modification of the His-tag (gluconoylation). A molecular mass of 26734.4±1.8 Da was obtained for RXRα. These results were in agreement with the molecular weights calculated from the known amino acid sequences.

The mass measurements of the noncovalent complexes were performed in ammonium acetate (200 mM; pH 8.0). Samples were diluted to 10 pmol/µl in the previous buffer and continuously infused into the ESI ion source at a flow rate of 3 µl/min through a Harvard syringe pump (Harvard Apparatus model 11). When studying non covalent complexes, a careful tuning of the parameters is necessary to transfer intact supramolecular complexes from the solution to the gas phase [58]. Attention must be specially paid to parameters related to the interface of the mass spectrometer. In particular, the hexapole RF value was set to 320 V and the capillary exit voltage (CE) was adjusted in each case. For the interaction analysis, ligands and SRC-1 NR2 peptide (676-CPSSHSSLTERHKILHRLLQEGSPS-700) were added to the proteins in a 5 fold molar excess.

### Isothermal titration calorimetry (ITC)

ITC measurements were performed at 30°C on a MicroCal ITC_200_ (MicroCal). Purified proteins were dialyzed extensively against the buffer used in the ITC experiments. The buffer contained 20 mM Tris pH 8.0, 200 mM sodium chloride. In a typical experiment, 1.5 or 2 µl aliquots of SRC-1 NR2 peptide (676-CPSSHSSLTERHKILHRLLQEGSPS-700) at 1.3 mM were injected at 0.5 µl.s^−1^ into a 20-50 µM RAR, RXR or RAR/RXR complexes solution (200 µl sample cell). In the complexes with ligands, ligand concentrations are in fivefold molar excess of NHR in all titrations. Equivalent amounts of ligand are added to both protein and peptide solutions and the ethanol concentrations are adjusted to 2% for all titrations. The delay between injections was 120 to 180 s to permit the signal to return to baseline before the next injection. ITC titration curves were analyzed using the software Origin 7.0 (OriginLab). Standard free energies of binding and entropic contributions were obtained, respectively, as Δ*G* = −*RT* ln(*K*_a_) and *T*Δ*S* = Δ*H* − Δ*G*, from the *K*_a_ and Δ*H* values derived from ITC curve fitting.

### Protein Data Bank Accession Number

The accession number for the coordinates of the complex reported in this article is 3A9E.

## Supporting Information

Figure S1 **RARα-** ***at*** **RA/RXRα-LG100754 LBDs is dimeric in solution.** (A) Comparison of the experimental SAXS curve of *at*RA-RAR/LG100754-RXR (blue cross) with the corresponding fits for the crystallographic model of the dimer (pink line), the tetramer (yellow line) and of the full-agonists dimer (cyan line). (B) Electron pair distribution [P(r)] function computed from the experimental SAXS data.
(EPS)

Figure S2 **Salt bridges at the heterodimer interface between RARα-** ***at*** **RA LBD and the RXRα-LG100754 LBD.** The RXR and RAR are shown by cartoon representation in green and cyan, respectively. Only residues forming salt bridges (dotted lines) are shown with oxygen atoms in red and nitrogen atoms in blue.
(EPS)

Figure S3 **Representative ITC titrations of SRC-1 NR2 peptide into RAR and RAR/RXR.**
(EPS)

Figure S4 **Docked complex between hRARα in agonist conformation and ligand LG100754 (in yellow).** Retinoic acid (in green) experimental position is indicated for comparison.
(EPS)

Figure S5 ***Fo – Fc*** **electron density omit map contoured at 3.0 σ for LG100754 (left) and** ***at*** **RA (right).**
(EPS)

Table S1 **Data collection and refinement statistics.**
(DOCX)

Table S2 **Binding parameters derived from ITC measurements for SRC-1 NR2 peptide to RAR and RAR/RXR.**
(DOCX)

## References

[1] Mark M, Ghyselinck NB, Chambon P (2009). Function of retinoic acid receptors during embryonic development.. Nucl Recept Signal.

[2] Mark M, Ghyselinck NB, Chambon P (2006). Function of retinoid nuclear receptors: lessons from genetic and pharmacological dissections of the retinoic acid signaling pathway during mouse embryogenesis.. Annu Rev Pharmacol Toxicol.

[3] Perissi V, Rosenfeld MG (2005). Controlling nuclear receptors: the circular logic of cofactor cycles.. Nat Rev Mol Cell Biol.

[4] Rosenfeld MG, Lunyak VV, Glass CK (2006). Sensors and signals: a coactivator/corepressor/epigenetic code for integrating signal-dependent programs of transcriptional response.. Genes Dev.

[5] Rochette-Egly C, Germain P (2009). Dynamic and combinatorial control of gene expression by nuclear retinoic acid receptors (RARs).. Nucl Recept Signal.

[6] Germain P, Iyer J, Zechel C, Gronemeyer H (2002). Co-regulator recruitment and the mechanism of retinoic acid receptor synergy.. Nature.

[7] Minucci S, Leid M, Toyama R, Saint-Jeannet JP, Peterson VJ (1997). Retinoid X receptor (RXR) within the RXR-retinoic acid receptor heterodimer binds its ligand and enhances retinoid-dependent gene expression.. Mol Cell Biol.

[8] Kersten S, Dawson MI, Lewis BA, Noy N (1996). Individual subunits of heterodimers comprised of retinoic acid and retinoid X receptors interact with their ligands independently.. Biochemistry.

[9] Chen JY, Clifford J, Zusi C, Starrett J, Tortolani D (1996). Two distinct actions of retinoid-receptor ligands.. Nature.

[10] DiRenzo J, Söderstrom M, Kurokawa R, Ogliastro MH, Ricote M (1997). Peroxisome proliferator-activated receptors and retinoic acid receptors differentially control the interactions of retinoid X receptor heterodimers with ligands, coactivators, and corepressors.. Mol Cell Biol.

[11] Aarnisalo P, Kim CH, Lee JW, Perlmann T (2002). Defining requirements for heterodimerization between the retinoid X receptor and the orphan nuclear receptor Nurr1.. J Biol Chem.

[12] Bourguet W, Vivat V, Wurtz JM, Chambon P, Gronemeyer H (2000). Crystal structure of a heterodimeric complex of RAR and RXR ligand-binding domains.. Mol Cell.

[13] Pogenberg V, Guichou JF, Vivat-Hannah V, Kammerer S, Pérez E (2005). Characterization of the interaction between retinoic acid receptor/retinoid X receptor (RAR/RXR) heterodimers and transcriptional coactivators through structural and fluorescence anisotropy studies.. J Biol Chem.

[14] Lala DS, Mukherjee R, Schulman IG, Koch SS, Dardashti LJ (1996). Activation of specific RXR heterodimers by an antagonist of RXR homodimers.. Nature.

[15] Mukherjee R, Jow L, Croston GE, Paterniti JR (1997). Identification, characterization, and tissue distribution of human peroxisome proliferator-activated receptor (PPAR) isoforms PPARgamma2 versus PPARgamma1 and activation with retinoid X receptor agonists and antagonists.. J Biol Chem.

[16] Schulman IG, Li C, Schwabe JW, Evans RM (1997). The phantom ligand effect: allosteric control of transcription by the retinoid X receptor.. Genes Dev.

[17] Lu HC, Revelli JP, Goering L, Thaller C, Eichele G (1997). Retinoid signaling is required for the establishment of a ZPA and for the expression of Hoxb-8, a mediator of ZPA formation.. Development.

[18] Billas IM, Iwema T, Garnier JM, Mitschler A, Rochel N (2003). Structural adaptability in the ligand-binding pocket of the ecdysone hormone receptor.. Nature.

[19] Egea PF, Rochel N, Birck C, Vachette P, Timmins PA (2001). Effects of ligand binding on the association properties and conformation in solution of retinoic acid receptors RXR and RAR.. J Mol Biol.

[20] Lafont V, Schaefer M, Stote RH, Altschuh D, Dejaegere A (2007). Protein-protein recognition and interaction hot spots in an antigen-antibody complex: free energy decomposition identifies “efficient amino acids”.. Proteins.

[21] Brelivet Y, Kammerer S, Rochel N, Poch O, Moras D (2004). Signature of the oligomeric behaviour of nuclear receptors at the sequence and structural level.. EMBO Rep.

[22] Vivat-Hannah V, Bourguet W, Gottardis M, Gronemeyer H (2003). Separation of retinoid X receptor homo- and heterodimerization functions.. Mol Cell Biol.

[23] Renaud JP, Rochel N, Ruff M, Vivat V, Chambon P (1995). Crystal structure of the RAR-gamma ligand-binding domain bound to all-trans retinoic acid.. Nature.

[24] Egea PF, Mitschler A, Rochel N, Ruff M, Chambon P (2000). Crystal structure of the human RXRalpha ligand-binding domain bound to its natural ligand: 9-cis retinoic acid.. EMBO J.

[25] Egea PF, Mitschler A, Moras D (2002). Molecular recognition of agonist ligands by RXRs.. Mol Endocrinol.

[26] Folkertsma S, van Noort PI, de Heer A, Carati P, Brandt R (2007). The use of in vitro peptide binding profiles and in silico ligand-receptor interaction profiles to describe ligand-induced conformations of the retinoid X receptor alpha ligand-binding domain.. Mol Endocrinol.

[27] Hashimoto Y, Miyachi H (2005). Nuclear receptor antagonists designed based on the helix-folding inhibition hypothesis.. Bioorg Med Chem.

[28] Pérez Santín E, Germain P, Quillard F, Khanwalkar H, Rodríguez-Barrios F (2009). Modulating retinoid X receptor with a series of (E)-3-[4-hydroxy-3-(3-alkoxy-5,5,8,8-tetramethyl-5,6,7,8-tetrahydronaphthalen-2-yl)phenyl]acrylic acids and their 4-alkoxy isomers.. J Med Chem.

[29] Ebisawa M, Umemiya H, Ohta K, Fukasawa H, Kawachi E (1999). Retinoid X receptor-antagonistic diazepinylbenzoic acids.. Chem Pharm Bull.

[30] Nahoum V, Pérez E, Germain P, Rodríguez-Barrios F, Manzo F (2007). Modulators of the structural dynamics of the retinoid X receptor to reveal receptor function.. Proc Natl Acad Sci USA.

[31] Koch SSC, Dardashti LJ, Hebert JJ, White SK, Croston GE (1996). Identification of the first retinoid X receptor homodimer antagonist.. J Med Chem.

[32] Lu J, Dawson MI, Hu QY, Xia Z, Dambacher JD (2009). The effect of antagonists on the conformational exchange of the retinoid X receptor alpha ligand-binding domain.. Magn Reson Chem.

[33] Lehmann JM, Jong L, Fanjul A, Cameron JF, Lu XP (1992). Retinoids selective for retinoid X receptor response pathways.. Science.

[34] Delescluse C, Cavey MT, Martin B, Bernard BA, Reichert U (1991). Selective high affinity retinoic acid receptor alpha or beta-gamma ligands. *Mol.*. Pharmacol.

[35] Xu J, Li Q (2003). Review of the in Vivo Functions of the p160 Steroid Receptor Coactivator Family.. Molecular Endocrinology.

[36] Pike AC, Brzozowski AM, Hubbard RE, Bonn T, Thorsell AG (1999). Structure of the ligand-binding domain of oestrogen receptor beta in the presence of a partial agonist and a full antagonist.. EMBO J.

[37] de Lera AR, Bourguet W, Altucci L, Gronemeyer H (2007). Design of selective nuclear receptor modulators: RAR and RXR as a case study.. Nat Rev Drug Discov.

[38] Otwinowski Z, Minor W (1997). Processing of X-ray diffraction data collected in oscillation mode.. Methods in Enzymology.

[39] Navaza J (1994). AMoRe-an automated package for molecular replacement.. Acta Crystallogr.

[40] Brünger AT, Adams PD, Clore GM, DeLano WL, Gros P (1998). Crystallography & NMR system: a new software suite for macromolecular structure determination.. Acta Crystallogr.

[41] Murshudov GN, Vagin AA, Dodson EJ (1997). Refinement of macromolecular Structures by the Maximum-Likelihood Method.. Acta Cryst.

[42] Afonine PV, Grosse-Kunstleve RW, Adams PD (2005). *CCP4 Newsl* 42, contribution 8.

[43] Jones TA, Zou JY, Cowan SW, Kjeldgaard M (1991). Improved methods for building protein models in electrondensity maps and the location of errors in these models.. Acta Crystallogr.

[44] Emsley P, Cowtan K (2004). Coot: model-building tools for molecular graphics.. Acta Crystallogr.

[45] Painter, J, Merritt EA (2006). Optimal description of a protein structure in terms of multiple groups undergoing TLS motion.. Acta Cryst.

[46] Laskowski RA, MacArthur MW, Moss DS, Thornton JM (1993). PROCHECK: a program to check the stereochemical quality of protein structures.. J Appl Cryst.

[47] Kleywegt GJ, Jones TA (1994). Detection, delineation, measurement and display of cavities in macromolecular structures.. Acta Cryst.

[48] Nicholls A Sharp KA, Honig B (1991). Protein folding and association: insights from the interfacial and thermodynamic properties of hydrocarbons.. Proteins.

[49] DeLano WL (2002). http://www.pymol.org.

[50] Potterton E, McNicholas S, Krissinel E, Cowtan K, Noble M (2002). The CCP4 molecular-graphics project.. Acta Cryst.

[51] Brunger AT, Karplus M (1988). Polar hydrogen positions in proteins: empirical energy placement and neutron diffraction comparison.. Proteins.

[52] Brooks BR, Bruccoleri RE, Olafson BD, States DJ, Swaminathan S (1983). Charmm: a program for macromolecular energy minimization and dynamics calculations.. J Comp Chem.

[53] Roessle MW et al (2007). Upgrade of the Small Angle X-ray scattering Beamline X33 at the European Molecular Biology Laboratory, Hamburg.. J Appl Crystallogr.

[54] Konarev PV, Volkov VV, Sokolova AV, Koch MHJ, Svergun DI (2003). PRIMUS: a Windows PC-based system for small-angle scattering data analysis.. J Appl Cryst.

[55] Guinier A (1939). Diffraction of x-rays of very small angles-application to the study of ultramicroscopic phenomena.. Ann Phys.

[56] Svergun DI (1992). Determination of the Regularization Parameter in Indirect-Transform Methods Using Perceptual Criteria.. J AppL Cryst.

[57] Svergun D, Barberato C, Koch MHJ (1995). CRYSOL – a Program to Evaluate X-ray Solution Scattering of Biological Macromolecules from Atomic Coordinates.. J Appl Cryst.

[58] Potier N, Rogniaux H, Chevreux G, Van Dorsselaer A (2005). Ligand-metal ion binding to proteins: investigation by ESI mass spectrometry.. Methods in Enzymology.

