# Supplementary material for: The “Phantom Effect” of the Rexinoid LG100754: Structural and Functional Insights
Source: PLoS One. 2010 Nov 30;5(11):e15119. doi: 10.1371/journal.pone.0015119 (PMC2994906; doi:10.1371/journal.pone.0015119)
Supplement: Table S1 — Data collection and refinement statistics. (DOCX) [file pone.0015119.s006.docx]

**Supplementary Table S1. Data collection and refinement statistics.**

| RARα−*at*RA/RXRα−LG100754 LBDs / TIF-2 CoA peptide | |
| --- | --- |
| *Data processing* |  |
| Resolution (Å) | 20-2.75 (2.85-2.75) |
| Crystal space group | P4_3_2_1_2 |
| Cell parameters (Å) | *a* = *b* = 105.3; *c* = 111.3 |
| Unique reflections | 15407 (1662) |
| Mean redundancy | 6.7 (7.6) |
| *R*_sym_ (%)^a^ | 9.7 (33.3) |
| Completeness (%) | 90.8 (99.6) |
| Mean *I*/*σ* | 15.2 (5.5) |
| Wilson *B* factor (Å^2^) | 54.3 |
| *Refinement* |  |
| Resolution (Å) | 20-2.75 |
| Number of non-hydrogen atoms |  |
| RAR-LBD | 1851 |
| RXR-LBD | 1644 |
| Coactivator peptide | 91 |
| Ligands | 51 |
| Water molecules | 115 |
| RMSD bond length (Å) | 0.002 |
| RMSD bond angles (˚) | 0.542 |
| *R*_cryst_ (%)^b^ | 20.1 |
| *R*_free_ (%)^c^ | 26.4 |
| Averaged *B* factor for  non-hydrogen atoms (Å^2^) |  |
| RAR-LBD | 53.3 |
| RXR-LBD | 59.1 |
| Coactivator peptide | 56.8 |
| *at*RA | 67.3 |
| LG100754 | 46.8 |
| Water | 53.5 |
| Ramachandran plot (%) |  |
| Core | 91.5 |
| Allow | 8.2 |
| Generous | 0.2 |

^a^ *R*_sym_ = 100 × Σ**_h_***_j_* |*I***_h_***_j_* – <*I***_h_**>| / Σ**_h_***_j_ I***_h_***_j_*, where *I***_h_***_j_* is the *j*th measurement of the intensity of reflection **h** and <*I***_h_**> is its mean value.

^b^ *R*_cryst_ = 100 × Σ||*F*_o_| – |*F*_c_|| / Σ|*F*_o_|, where |*F*_o_| and |*F*_c_| are the observed and calculated structure factor amplitudes, respectively.

^c^ Calculated using a random set containing 5% of observations that were not included throughout refinement [Brünger A. T., (1992) The Free R Value: a Novel Statistical Quantity for Assessing the Accuracy of Crystal Structures, *Nature* **355**, 472-474].
