# Supplementary material for: The “Phantom Effect” of the Rexinoid LG100754: Structural and Functional Insights
Source: PLoS One. 2010 Nov 30;5(11):e15119. doi: 10.1371/journal.pone.0015119 (PMC2994906; doi:10.1371/journal.pone.0015119)
Supplement: Table S2 — Binding parameters derived from ITC measurements for SRC-1 NR2 peptide to RAR and RAR/RXR. (DOCX) [file pone.0015119.s007.docx]

**Supplementary Table 2.**

Binding parameters derived from ITC measurements for SRC-1 NR2 peptide to RAR and RAR/RXR.

Kd (µM) ΔH (cal/mol) ΔS (cal/mol/K)

RAR apo 9.0 ± 0.4 -5862 ± 240 3.76

RAR-agonist 1.4 ± 0.1 -3129 ± 19 16.2

RAR-LG100754 1.6 ± 0.2 -7695 ± 479 -3.39

RAR-LG100754/RXR-LG100754 4.5 ± 0.6 -3755 ± 98 12.1
